# Compatibility between Legumes and Rhizobia for the Establishment of a Successful Nitrogen-Fixing Symbiosis

**DOI:** 10.3390/genes9030125

**Published:** 2018-02-27

**Authors:** Joaquín Clúa, Carla Roda, María Eugenia Zanetti, Flavio A. Blanco

**Affiliations:** Instituto de Biotecnología y Biología Molecular, Facultad de Ciencias Exactas, Universidad Nacional de La Plata, Centro Científico y Tecnológico-La Plata, Consejo Nacional de Investigaciones Científicas y Técnicas, 1900-La Plata, Argentina; joaquinclua@biol.unlp.edu.ar (J.C.); croda@biol.unlp.edu.ar (C.R.); ezanetti@biol.unlp.edu.ar (M.E.Z.)

**Keywords:** nodulation, legume, rhizobia, soil bacteria

## Abstract

The root nodule symbiosis established between legumes and rhizobia is an exquisite biological interaction responsible for fixing a significant amount of nitrogen in terrestrial ecosystems. The success of this interaction depends on the recognition of the right partner by the plant within the richest microbial ecosystems on Earth, the soil. Recent metagenomic studies of the soil biome have revealed its complexity, which includes microorganisms that affect plant fitness and growth in a beneficial, harmful, or neutral manner. In this complex scenario, understanding the molecular mechanisms by which legumes recognize and discriminate rhizobia from pathogens, but also between distinct rhizobia species and strains that differ in their symbiotic performance, is a considerable challenge. In this work, we will review how plants are able to recognize and select symbiotic partners from a vast diversity of surrounding bacteria. We will also analyze recent advances that contribute to understand changes in plant gene expression associated with the outcome of the symbiotic interaction. These aspects of nitrogen-fixing symbiosis should contribute to translate the knowledge generated in basic laboratory research into biotechnological advances to improve the efficiency of the nitrogen-fixing symbiosis in agronomic systems.

## 1. Introduction

The economic and ecological importance of legumes is evidenced by the high number of species that are cultivated and commercialized, as well as by their ability to obtain nitrogen from a symbiotic interaction with soil bacteria known as rhizobia. This family of flowering plants includes species of agronomic importance such as common bean (*Phaseolus vulgaris*), alfalfa (*Medicago sativa*), soybean (*Glycine max*), pea (*Pisum sativum*), and lentil (*Lens culinaris*), etc. Their unique capacity to establish a nitrogen-fixing symbiosis among crops is crucial to alleviate the usage of synthetic fertilizers in agronomic systems. Nitrogen fertilization is extremely expensive and generates ecological risks such as water eutrophication and emission of atmospheric greenhouse gases that contribute to global warming. Biological nitrogen fixation is an ecologically safe alternative but is restricted to the symbiotic interaction of a small group of plants (mainly legumes and actinorhizal plants) with nitrogen-fixing microorganisms. In root legume symbiosis, the interaction is based on the capacity of rhizobia to convert atmospheric N_2_ into chemical forms that can be incorporated into the plant metabolism. During this interaction, bacteria are internalized as endosymbionts within the cells of a post-embryonic root organ, the nodule. Formation of functional nitrogen-fixing nodules depends on two separate, but highly coordinated processes, the infection by rhizobia and the organogenesis of the nodule, which occur in the epidermal and cortical cell layers of the root, respectively. In some legume species, infection takes place through a host-derived tubular compartment that growths inward in the root trichoblast (i.e., epidermal cells with root hairs), known as the infection thread (IT). Physiological changes start with the re-directioning of the root hair polar growth produced by bacteria attachment to the surface, generating a shepherd hook structure that entraps the bacterial microcolony to form an infection pocket. From this point, the root hair plasma membrane invaginates and starts to elongate toward the interior of the epidermal cell [1]. Concomitantly, nodule organogenesis is initiated in the cortex, where cells have been re-activated for mitosis to form the nodule primordia. Infection threads grow toward the interior of the root to reach the nodule primordia, and bacteria are internalized and allocated in organelle-like structures, called symbiosomes, where they differentiate into bacteroids and begin to fix atmospheric nitrogen by the activity of the nitrogenase enzyme complex. Even though this infection mechanism is the most studied and representative of many important crop legumes, other species are infected in alternative ways that reflect the diversity of habitats and lifestyles of legume plants, including crack-entry and intercellular infection [1]. 

**Figure 1:**
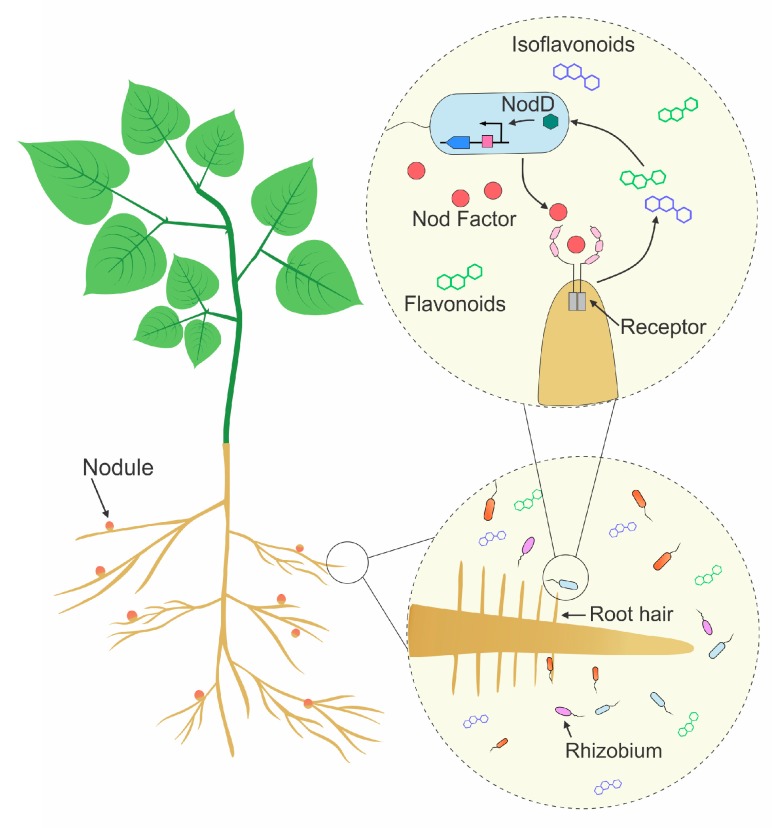
Signal exchange in the legume-rhizobia interaction. Flavonoids and isoflavonoids secreted by legume roots activate the Nodulation protein D (NodD) transcription factor on compatible rhizobia inducing the transcription of *nod* genes, which are required for the synthesis of Nod factors. Nod factors are perceived by receptors present in the plasma membrane of root cells, triggering the signaling pathway required for the development and infection of the nodule, where bacteria are allocated, and nitrogen fixation occurs.

The symbiotic interaction is initiated after an initial exchange of signals: when nitrogen present in the soil is scarce, legumes exude a series of phenolic compounds into the rhizosphere, mainly flavonoids and isoflavonoids (Figure 1) [2]. These molecules are sensed by rhizobia, activating the transcriptional regulator Nodulation protein D (nodD), which in turn triggers the transcription of genes required for the synthesis of the Nod factor [3]. Nod factors are lipochitooligosaccharides secreted by rhizobia and perceived by receptors present in the plasma membrane of root cells. Perception of Nod factors is necessary and usually sufficient to provoke molecular and physiological responses in the plant, such as accumulation of early nodulin transcripts (i.e., genes induced in the plant during nitrogen-fixing symbiosis), root hair curling and the formation of the IT, where bacteria can divide and progress toward the cortical cells [4]. Even though this initial perception occurs at the epidermis, continuous signaling at later stages is important for progression of infection and bacterial release [5,6,7,8].

Many molecular components of the signaling and response of plants have been unveiled, disclosing a signal transduction pathway known as the Nod pathway, which shares many elements with the signaling pathway of a more ancient symbiosis established by most plants with mycorrhizal fungi [9]. The Nod pathway is initiated by the perception of Nod factors by Nod factor receptors, which contain a cytoplasmic kinase, a transmembrane domain and an extracellular region with two or three lysin motif (LysM) domains [10,11,12,13,14]. A receptor-like kinase with extracellular leucine-rich repeats, known as symbiosis receptor-like kinase (SYMRK), is also necessary for nodule formation [15,16,17]; however, its ligand is still unknown. Cleavage of the SYMRK extracellular domain promotes its association with the Nod factor receptor 5 (NFR5). Downstream transmission of Nod factor receptors requires the oscillation of calcium concentration within and around the nucleus [18,19]. Potassium-permeable channels [20,21,22] and cyclic nucleotide-gated channels located at the nuclear envelope [23] are required for activation of calcium oscillations. Within the nucleus, calcium oscillations are decoded by a calcium-calmodulin dependent protein kinase (CCaMK) [24] that associates with and phosphorylates the coiled-coil protein CYCLOPS [25], initiating a cascade of transcription factors that results in the transcriptional reprogramming of the cells committed for symbiosis. 

Most of the advances in the area came from the study of two model legumes selected by the scientific community, *Medicago truncatula* and *Lotus japonicus*. Both plants are representatives of forage species, but they form different types of nodules; whereas *M. truncatula* displays cylindrical indeterminate nodules that retain a persistent meristem, *L. japonicus* forms spherical nodules, which cease cell division very early in nodule development, thus the nodules grow by cell enlargement. Despite the enormous progress achieved in the understanding of the molecular mechanisms involved in the root legume symbiosis, these advances have had a poor impact in the field, where nitrogen fixation is considered suboptimal in terms of the theoretical capacity of the process. This is mainly due to a poor understanding of the ecological aspects of the interaction that should help to explain why indigenous populations of rhizobia have a strong capacity to compete and occupy nodules as compared with efficient strains added to the soils in the form of inoculants.

In this review, we will introduce and discuss aspects of the symbiotic interaction that can shed some light on how nitrogen-fixing symbiosis works in a complex ecological system, the soil, with emphasis on the genetic bases of the legume-rhizobia compatibility that control the ability of the plant to select the rhizobial strains that better fulfill plant nitrogen requirements.

## 2. Legume-Rhizobia Interaction in a Community Context

The plant signal transduction pathway and molecular responses underlying the legume-rhizobia symbiosis were elucidated mainly through the study of plant-host binary interactions in gnotobiotic systems, but in natural ecosystems, plants encounter diverse communities of microorganisms that affect plant growth and health through mutualistic, commensalistic and parasitic plant-microorganism relationships. Characterization of this community, known as the plant microbiota, is important to contextualize the molecular mechanisms by which legumes recognize rhizobia within the bacterial communities of the soil and root microbiota.

**Figure 2:**
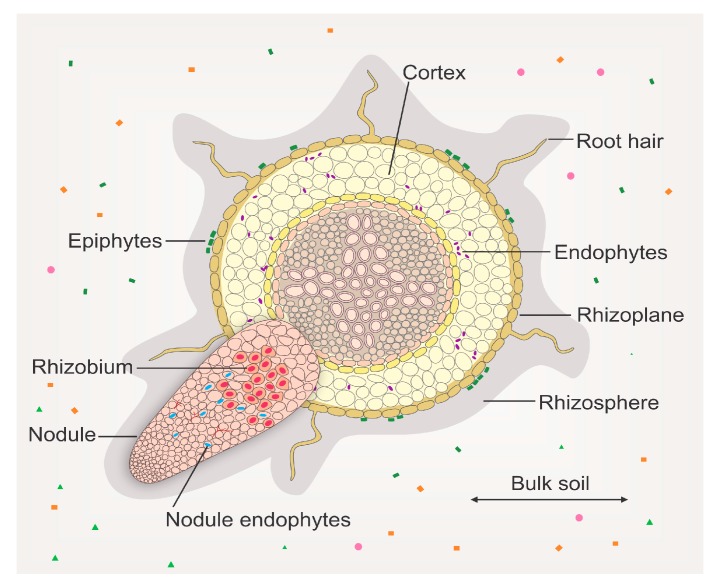
The complexity of bacterial root microbiota. The bacterial community decreases in complexity from the rich soil microbiota to the rhizosphere, the rhizoplane, the endosphere, and the nodule. The rhizosphere is colonized by a subset of the bulk soil community, and the rhizoplane hosts epiphytes that are firmly attached to the root surface. The endosphere (root interior) is inhabited by endophytes. The root nodule is the habitat of symbiotic nitrogen-fixing bacteria, known as rhizobia, and also harbors a diversity of non-fixing bacteria called nodule endophytes. Infection threads can be coinhabited by endophytes and rhizobia.

A typical gram of soil contains between 10^6^–10^9^ bacterial cells representing more than 10,000 different species [26]. The application of high throughput sequencing technologies to metagenomic studies has enabled the quantitative description of bacterial communities of soil and belowground compartments of plants [27,28]. Two pioneer papers describing the *Arabidopsis thaliana* microbiome agreed that host-associated bacterial communities are not stochastically formed, but show defined phylogenetic structures that vary across the soil-root continuum: bulk soil, rhizosphere (narrow region of soil surrounding roots), rhizoplane (harbors microorganisms firmly adhered to the root surface, termed epiphytes) and endosphere (the habitat inside plant organs that host endophytes) (Figure 2) [29,30]. Lundberg and co-workers [30] used 454 pyrosequencing to sequence bacterial 16S ribosomal RNA (rRNA) from 8 *A. thaliana* accessions grown in 2 soils from different locations and analyzed the microbiota of bulk soil, rhizosphere and the endophytic compartment (EC), resulting in 1248 ribotyped (bacteria classification based on rRNA-based phylogenetic analyses) samples. The authors showed that 256 operational taxonomic units (OTUs) were significantly different in the rhizosphere and the EC as compared to soil, 164 of which were enriched in EC and 32 were depleted, defining the *A. thaliana* endophytic microbiome. The EC microbiome was composed principally by Actinobacteria, Proteobacteria and Firmicutes, and was depleted of Acidobacteria, Gemmatimonadetes and Verrucomicrobia [30]. Bulgarelli et al. reported that the dominant phyla in *A. thaliana* roots were Proteobacteria, Bacteroidetes and Actinobacteria [29]. Similar results were obtained in other plant species ranging from eudicots to monocots [28]. They all showed that bacteria plant microbiota is principally composed of a few phyla, Proteobacteria, Actinobacteria, Bacteroidetes and Firmicutes, and that there is a selective gradient in microbiota diversity across the soil-root continuum, being the bulk soil microbiota the most complex and the endosphere the most selective. Collectively, these studies suggest that plants actively influence the function and composition of their microbiota through rhizodeposition, water and mineral release and the action of the host immune system.

Although the root nodule is commonly seen as an exclusive habitat of symbiotic nitrogen-fixing bacteria, it hosts a diverse population of non-nodulating bacteria called non-rhizobia endophytes [31], nodule endophytes [32], or nodule-associated bacteria [33] belonging to diverse genera, i.e., *Azospirillum*, *Burkholderia*, *Klebsiella*, *Arthrobacter* and *Bacillus* (for an exhaustive review, see [34]). A recent study described the nodule microbiome of two cowpea genotypes cultivated in two different soil. As expected, *Bradyrhizobium* was the predominant genus, but high bacterial diversity was found associated with nodules, mainly OTUs related to the genera *Enterobacter*, *Chryseobacterium*, *Sphingobacterium* and unclassified *Enterobacteriacea*. Soil type, and to a lesser extent plant genotype, influenced the composition of these bacterial communities [35]. Functional studies conducted in *L. japonicus* in association with its cognate partner *Mesorhizobium loti* and endophytic bacteria, both expressing fluorescent proteins, showed that the infection threads induced by *M. loti* can be coinhabited by the endophytes *Herbaspirillum* sp. B501, *Rhizobium giardinii* sp. 129E, *Burkholderia* sp. KAW25 (KAW25) and *Rhizobium mesosinicum* strain KAW12 (KAW12), and that particularly KAW25 and KAW12 can employ the infection thread to reach the nodule and multiply. Moreover, it was observed that nodule infection by KAW12 is dependent on a functional Nod factor-induced infection pathway and that exopolysaccharides (EPS) are critical for symbiotic and endophytic nodule colonization. When the mutant strain *M. loti R7A exoU*, which synthesizes incompatible EPS and induces aberrant ITs, was co-inoculated with KAW12, the arrested infection process was rescued and an increased frequency of KAW12 nodule colonization was observed. On the other hand, the co-inoculation of the wild type strain of *M. loti* and an *eps* mutant of KAW12 deficient in EPS synthesis resulted in lower colonization frequency by the KAW12 mutant with respect to the wild type strain. These results strongly suggest that *M. loti* EPS signatures provide an advantage to the microsymbiont over the endophyte [36].

Another interesting twist in the comprehension of the relationship between root nodule symbiosis and the root plant microbiome was revealed by the study conucted by Zgadzaj et al. [37]. Firstly, the authors compared the structure of bacterial communities present in uninoculated roots and nodules of *L. japonicus* wild type plants, the rhizosphere, and unplanted soil. The results revealed that each fraction has a distinctive bacterial community that decreases in complexity from the rich soil microbiota to the rhizosphere, the root, and the nodule. They found that 12 out of a total of 1834 OTUs were nodule-enriched and that the nodule bacterial community was dominated by Rhizobiales, mainly due to the selective enrichment of *Mesorhizobium* members, although the presence of Burkholderiales, Flavobacteriales, Pseudomonadales, and Actinobacteridae was also detected, showing that *L. japonicus* nodules are not only colonized by symbiotic bacteria. Interestingly, the same experimental procedure was applied to *L. japonicus* mutants with respect to the *nfr5*-2 and *nfr5*-3, Nodule inception (*nin*-2) and Lotus histidine kinase 1 (*lhk1*-1) genes, which are required for the rhizobial infection and the formation of functional nodules. The results showed that in the four mutant backgrounds, the bacterial communities associated with roots and rhizosphere were similar to each other, but significantly different from the communities of wild type plants. The number of enriched OTUs in the rhizosphere of the symbiotic mutant plants increased by a factor of eight compared with the wild type, while there was a strong reduction in the number of root-enriched OTUs (105 in wild type versus 28 in mutants). Strikingly, the bacterial community shifts observed in mutants were similar when soil was supplemented with KNO_3_, suggesting a direct role of the symbiotic signaling pathway in the establishment of host root and rhizosphere microbiota rather than an indirect consequence of nitrogen fixation. This may imply that root-associated microbiota directly use the nodulation signaling pathway and/or cooperate with rhizobia through microbe-microbe interactions to successfully colonize roots [38].

The implementation of high throughput sequencing technologies to the analysis of plant microbiota highlighted the diversity of bacterial communities present in the rhizosphere, the interior of the root and, more specifically, the nodule. The occurrence of a selective gradient from bulk soil to the inner tissues of the root points toward an active role played by the plant innate immunity system in shaping the root microbiota, opening the entrance to rhizobial infection within a rich bacterial community, while closing it to pathogen invasion. This apparent paradox is an active research field that houses the answer to fully understand legume-rhizobia symbiosis success.

## 3. Distinguishing Friends from Enemies

Plants and bacteria can establish different types of ecological interactions, ranging from mutually beneficial (symbiosis or mutualism) to those detrimental for the plant (pathogenic), with a majority of relationships in which none of the partners are affected (commensalism). Symbiotic interactions were established very early and were probably key in the transition of plants from aquatic to terrestrial habitats. While plants evolved to interact with bacteria and fungi to overcome nutrient scarcity in this new environment, pathogenic interactions imposed the selective pressure that resulted in the development of sophisticated mechanisms that allowed them to recognize and respond to the diversity of microorganisms present in the soil. 

Plants, unlike animals, lack specialized cell lines with functions in pathogen surveillance and detection, or an adaptive immune system. Instead, each plant cell is able to trigger an innate immune response based on the recognition of evolutionary conserved microbial molecules termed microbial- or pathogen-associated molecular patterns (MAMPS or PAMPs). This recognition requires plant plasma-membrane receptors termed pattern recognition receptors (PRRs). The immune response initiated by these receptors is designated as PAMP-triggered immunity (PTI) and represents the first tier of the plant immune system. Successful pathogens acquired the ability to overcome PTI delivering suppressors of the defense response (called effectors) into the cytoplasm of the host, resulting in effector-triggered susceptibility (ETS). On the other hand, resistant plants have evolved a second tier of defense called effector-trigger immunity (ETI) to deal with adapted pathogens, which relies on the specific recognition of one effector by the product of one disease resistance (R) gene, most of them, intracellular receptors with nucleotide binding sites and leucine rich repeat domains (NBS-LRR receptors) [39,40,41]. Common defense responses such as oxidative burst, phytoalexin production, hormonal changes, and transcriptional reprogramming are triggered during PTI and ETI, although ETI responses are more prolonged and robust [42]. 

**Figure 3:**
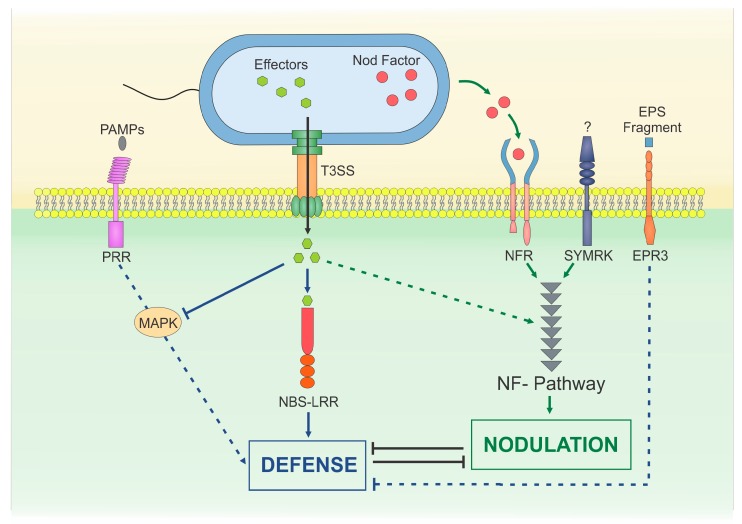
The interplay between nodulation and defense signaling pathways. Plants perceive bacterial molecules (pathogen-associated molecular patterns, PAMPs) using pattern recognition receptors (PPRs) that activate mitogen activated kinase (MAPK) cascades that trigger host defense responses. Adapted pathogens use the type III secretion system (T3SS) to deliver effector proteins into the cytosol of host cells. Bacterial effectors can inhibit the MAPK cascade, leading to suppression of host defenses. In some plants varieties, these effectors are recognized by nucleotide binding site- leucine rich repeat domains (NBS-LRR) receptors, which trigger a second tier of host defense responses. Recognition of Nod factors produced by compatible rhizobia by specific receptors (NFR) triggers a signaling cascade leading to nodulation (NF-Pathway). Rhizobial effectors can also promote nodulation by directly activating the NF-Pathway. The symbiosis receptor-like kinase (SYMRK) is also necessary for nodule formation, but the nature of its putative ligand is unknown. In a second stage of rhizobia recognition, exopolysaccharides (EPS) produced by rhizobia are perceived by exopolysaccharide protein receptor 3 (EPR3), inactivating the defense signaling pathway through unknown mechanisms.

Receptors of pathogen-derived molecules contain different recognition extracellular domains, mainly LRR and lectin-binding domains, able to recognize proteins/peptides and secreted carbohydrates, respectively, present on the surface of pathogens or generated by the action of hydrolytic enzymes on the plant cell wall. Chitin-derived molecules are important in the signaling response to pathogenic fungi whose cell walls are susceptible to degradation by plant chitinases. Chitooligosaccharide (CO) and lipochitooligosaccharide (LCO) molecules are also important for mycorrhiza [43,44] and root nodule symbiosis signaling [45,46]. Receptors for Nod factors and rhizobia EPS contain extracellular LysM domains [10,11,12,13,47,48,49], which are also a common motif present in PRRs. A recent report described that distinct members of the LysM receptor families of *L. japonicus* and *M. truncatula* are involved in CO and LCO perception, initiating either the defense response or the symbiotic program [50]. These results suggest that both signal transduction pathways are independent and can exert a negative effect on each other (Figure 3). 

It is assumed that during rhizobia-legume symbiosis, plant defense must be suppressed or avoided by rhizobia to successfully reach the nodule through infection threads and eventually be intracellularly accommodated. Time-course transcriptomic experiments conducted in soybean (*G. max*) [51], *M. truncatula* [52] and *L. japonicus* [53] support this hypothesis. These studies consistently revealed that there is a transient induction of defense-related genes at early stages of the interaction with rhizobia that returns to basal levels or is even repressed at later stages. For example, Lohar et al., reported a transcriptomic study comparing *Sinorhizobium meliloti-*inoculated versus uninoculated *M. truncatula* roots at 1, 6, 12, 24, 48 and 72 h post inoculation (hpi) [52]. A clustering analysis based on differential expressed genes at all time points identified four stages corresponding to transcriptional changes in response to rhizobia: stage I (1 hpi), stage II (6 and 12 hpi), stage III (24 to 48 hpi) and stage IV at 72 hpi. The *defense and disease response* category were significantly overrepresented in the list of genes upregulated at stage I. However, this group was overrepresented among genes downregulated at later stages of the interaction with the highest suppressed at stage III, which corresponds to the time of infection thread formation in the curled root hairs [52]. 

The bacterial peptide flg22, which correspond to the N-terminal region of the flagellum protein flagellin, acts as a potent elicitor of immunity in most plant species. However, the N-terminal region of rhizobial flagellin is exceptionally divergent and lacks amino acids that are important for immunogenicity, suggesting that rhizobia can evade recognition of this molecular pattern [54,55]. The interplay between symbiosis and defense was also evidenced by functional analysis. *L. japonicus* roots treated with the elicitor flg22 or a *M. loti* cell suspension showed induced defense responses, such as an increase in ethylene production, mitogen activated kinase (MAPK) activation and induction of canonical defense genes. Interestingly, flg22 treatment caused a delay in nodulation and a reduction in nodule number both in wild type *L. japonicus* plants and in the gain-of-function mutant of the spontaneous nodule formation 1 (*snf1*) allele, which encodes a constitutively active version of CCaMK that triggers the development of nodules in the absence of rhizobia. However, when flg22 was applied four weeks after rhizobia inoculation, no differences in nodule number were detected [56]. Notably, a suspension of flagellin isolated from *M. loti* failed to induce ethylene accumulation or MAPK activation, supporting the notion that rhizobial flagellin is not a PAMP [54]. Consistent with these results, inoculation of *M. truncatula* with the pathogenic bacteria *Pseudomonas syringae* pv. tomato strain DC3000 (Pst DC3000) or the symbiont *S. meliloti* strain Sm2011 (Sm2011) alone led to the induction of defense responses. However, when co-inoculated, Sm2011 suppressed the defense responses triggered by Pst DC3000 and conversely, Pst DC3000 reduced the number of infection threads and nodules formed by Sm2011 and the expression of the symbiosis marker genes *NIN* and Nodule Pectate Lyase (*NPL*) [57]. Together, these results suggest that defense responses have an antagonistic effect in rhizobial infection and nodule organogenesis, but once the symbiosis is established, the symbiotic pathway overcomes the defensive response. It is also clear that after a transient induction of defense responses, there is a repression of this response that allows rhizobial infection. However, it is still not clear how these two signaling pathways are interrelated. An interesting hypothesis proposes that the rhizobial symbiosis has evolved from pathogenic interactions [58], which is strongly supported by the shared molecular machinery and mechanisms used by plants and bacteria to establish both types of interactions. Nod Factors and some PAMPs are both recognized by plasma membrane receptors containing LysM extracellular domains. Bacterial effectors delivered into the plant cell cytoplasm by type III (T3SS), IV (T4SS) and VI (T6SS) secretion systems also play dual roles in pathogenesis and symbiosis [59,60]. Similar to pathogens, rhizobia possess molecular machinery to transfer proteins into the cytosol of the host cells, including T3SS, T4SS, and T6SS secretion systems. The presence of these secretion systems and/or effector proteins delivered through them is a key determinant of host specificity [61,62,63,64]. Although little is known about their molecular functions during symbiosis, some reports suggest a role in the suppression of plant defense responses; however, these effectors can be recognized by the surveillance machinery and can activate ETI, restricting the establishment of symbiosis. In line with this, the analyses of rhizobia harboring mutations that abolish the secretion system formation revealed both positive and negative effects on host range. For example, abolition of T3SS-dependent protein secretion in *Sinorhizobium fredii* NGR234 produces different phenotypes depending on the plant genotype; it has no significant effect on *Vigna unguiculata*, whereas it has a negative effect on *Lablab purpureus*, *Flemingia congesta* and *Tephrosia vogelii* when compared to plants inoculated with the wild type strain. On the other hand, a positive effect was shown in *Crotalaria juncea*, *Pachyrhizus tuberosus* and *Leucaena leucocephala* [65]. The relevance of T3SS was further evidenced by Okazaki et al., 2013 [66]. The authors reported that *Bradyrhizobium elkanii* USDA61 is able to infect and nodulate *nfr1* mutant plants of soybean in a T3SS-dependent manner [66,67]. Moreover, a *nodC* mutant of *B. alkanii* (BEnodC), which is unable to synthesize Nod factors, is also able to nodulate *nfr1* plants, although the nodulation efficiency was lower than the wild type strain. This result suggests a synergistic effect of the Nod factor and T3SS signaling in the establishment of a successful symbiosis. To dissect plant transcriptional responses exhibited by *B. elkanii* T3SS, *nfr1* soybean plants were inoculated with USDA61 or the T3SS mutant, and the root transcriptomes were compared using microarray analysis. Interestingly, T3SS-derived signaling activates several symbiosis marker genes known to be upregulated by the perception of compatible Nod factors, including *NIN* and early nodulin 40 (*ENOD40*) [66]. Identification of the effector/s delivered by the T3SS that hijacks soybean symbiosis signaling remains elusive.

Few effector proteins have been reported as important for nodulation, either with positive or negative effects. Interestingly, some of these effectors were reported to be involved in the modulation of plant immune signaling [59]. The NopM (nodulation outer protein M) effector of *Rhizobium* sp. NGR234 is an E3 ubiquitin ligase with LRRs that is required for the efficient nodulation of *L. purpureus*. Plants inoculated with NGR234 carrying a point mutation in *NopM* that impairs its enzymatic activity showed a reduced number of nodules compared to the wild type strain [68,69]. However, no difference, or even the opposite effect was observed in other plant species, such as *Tephrosia vogelii* and *Pachyrhizus tuberosus*, respectively [69]. The over-expression of NopM in *Nicotiana benthamiana* nearly completely abolished the flg22-induced reactive oxygen species (ROS) burst, suggesting that NopM blocks ROS-associated defense responses [68]. The NGR234 effector protein NopL has a positive effect on nodule number formation in *F. congesta* and antagonizes nodule senescence in *P. vulgaris*. When expressed *in planta*, NopL is targeted to the nucleus, where it interacts and is phosphorylated by the salicylic acid-induced protein kinase (SIPK). The NopL over-expression also prevents full induction of pathogenesis-related (PR) defense proteins [70,71,72,73]. 

On the other hand, plant genes that restrict nodulation have been associated with the recognition of rhizobial effector proteins. The soybean allelic genes *Rj2* and *Rfg1* encode an R protein of the Toll-interleukin receptor/nucleotide-binding site/leucine-rich repeat (TIR-NBS-LRR), which restricts nodulation by *Bradyrhizobium japonicum* (USDA122) and *S. fredii* (USDA257), respectively [74]. The introduction of the *Rj2* allele in an *rj2/rj2* background resulted in a complete block of nodulation by the compatible strain USDA122, whereas post-transcriptional gene silencing of *Rj2* in an *Rj2* background enabled nodulation by USDA122. This suggests that an unidentified rhizobial effector is recognized by this R protein, triggering ETI and restricting nodulation. Consistently, a T3SS mutant of USDA257 gained the ability to nodulate soybean plants carrying an *Rfg1* allele [74]. Another Rj soybean gene called *Rj4*, restricts nodulation by many strains of *B. elkanii* [75]. The *Rj4* gene has been reported to encode a thaumatin-like protein, that when mutated by the CRISPR/Cas9 system, enabled nodulation by the incompatible rhizobia *B. elkanii* (USDA61) [76]. On the bacterial side, restriction of the symbiotic interaction is dependent on a functional T3SS [77]. Recently, specific effectors that determine the incompatibility between USDA61 and the *Rj4* soybean BARC2 cultivar have been identified, including a cysteine protease with homology to xopD, a type 3 effector protein of *Xanthomonas campestris* [78]. Inoculation with USDA61 induces the accumulation of H_2_O_2_ and salicylic acid at early stages of the interaction in BARC2 roots. The transcriptomic analysis of BARC2 roots inoculated with USDA61 or with a T3SS mutant revealed that defense-related genes were induced by *B. elkanii* T3SS [78]. Together, these results suggest that rhizobial effector proteins are double edged swords in plant-rhizobia recognition: in compatible plant genotypes, effectors promote nodulation by suppression of plant defense responses, but when recognized by R proteins of incompatible hosts, ETI is triggered and rhizobial infection is blocked.

## 4. Recognition and Signaling Events for the Establishment of a Compatible Interaction between Legume and Rhizobia

As plants are surrounded by a diversity of microorganisms present in the soil, they have evolved sophisticated recognition mechanisms that allow them to discriminate not only between benefic and potentially pathogenic bacteria but also between compatible and incompatible rhizobia. In the legume-rhizobia interaction, molecules produced by the bacterium, mainly the Nod factor, trigger a signal transduction pathway in the root cells that activates bacterial infection and nodule organogenesis, which are coordinated in time and space. Nod factors are the main determinants of host specificity. Each rhizobium species produce a particular mix of Nod factors characterized by chemical substitutions at certain positions that give specificity to the signaling. It has been shown that changes in the extracellular domain of the Nod Factor receptors are sufficient to extend host specificity between two species of the *Lotus* genus [79]. 

Other molecules, such as lipopolysaccharides (LPS), EPS and secreted proteins, sometimes referred as secondary signals, have been proposed to participate in the suppression of defense responses, allowing rhizobia to progress toward cortical cells without triggering the hypersensitive response or other defense responses triggered by pathogenic bacteria [80,81]. It has been suggested that the suppression of defense responses is important not only at early stages of the infection process, but also in mature nodules to avoid the interruption of the infection, similarly to that observed in animal systems, where the presence of LPS and secreted proteins are required to maintain a chronic infection [38]. Mutant strains impared in the production of these molecules has been used to elucidate their biological function during symbiosis. For example, mutants affected in EPS biosynthesis are unable to trigger the formation of infection threads [82,83]. A receptor like-kinase that binds the EPS from *M. loti* has recently been identified in *L. japonicus* and designated as EPR3 [47]. Expression of *EPR3* is induced by Nod factor perception, indicating that a two-step recognition mechanism controls rhizobial infection. The second step depending on EPS recognition could help to discriminate between compatible and incompatible bacteria. 

Lipopolysaccharides are composed of a core oligosaccharide, a lipidic substitution and a polymer (known as the O-antigen) that is exposed on the surface of the cell wall. As in the case of EPS mutants, inoculation with strains affected in the synthesis of lipopolysaccharides leads to abnormalities in the infection process [84,85,86]. Moreover, a mutant of *Rhizobium* sp. IRBG74 affected in the synthesis of the LPS showed defects not only in the nodulation of the aquatic legume *Sesbania cannabina*, but also in its capacity to promote the growth of rice as an endophyte [87]. It has been proposed that LPSs are involved in the suppression of the defense responses triggered by pathogens, a prerequisite for the progression of rhizobia to the interior of the root [88,89]. A similar function has also been suggested for Nod factors [90,91] and EPS [92,93]. Besides the proposed role for signal molecules in defense suppression, nodulation phenotypes are highly variable depending on the host and the particular mutation in the rhizobial molecule.

Compatibility between legumes and rhizobia can also be established at late stages of symbiosis, during the infection process and even in the nodule. Plants produce cystein-rich peptides that are secreted to control differentiation of rhizobia. In indeterminate nodules, which contain a persistent meristem, bacteroid differentiation includes endoreplication and cell expansion, leading to a developmental stage known as terminal differentiation since these bacteria can no longer proliferate on culture media. This is under the combined control of host peptides and bacterial peptidases able to cleave them [94,95,96]. Recently, it has been shown that members of the nodule-specific cysteine-rich (NCR) family of peptides can control bacterial proliferation inside the nodule, leading to incompatibility between strains of rhizobia in combination with plant genotypes according to the presence of host *NCR* alleles [97,98].

Together, the exchange of signals between both symbionts shows how the compatibility between plants and rhizobia is established at early stages of the interaction and maintained later on to avoid proliferation of inefficient versions of the microsymbiont. 

## 5. Transcriptional Reprogramming Required for a Compatible Root Nodule Symbiosis

Perception of rhizobia by legume roots leads to the activation of several transcription factors, including members of the nin-like [99,100], ethylene response factor/Apetala2 (ERF/AP2) [101,102,103], GRAS [104,105] and Nuclear Factor Y (NF-Y) families [106,107,108,109], which initiate the transcriptional reprogramming of symbiotic cells. In addition, transcriptomic studies at global scale, including microarray and RNAseq experiments, have shown changes in mRNA levels of transcription factors of many families, such as Homeodomain-Leucine Zipper (HD-ZIP), WRKY, basic Leucine Zipper (bZIP), etc. Initial changes in these transcriptional regulators lead to massive changes in the steady-state levels of cellular mRNAs belonging to different functional categories, including metabolism, hormonal balance, stress responses, translational regulation, etc. Several studies have described in detail the dynamic changes occurring in the root at early time points of the interaction, as well as at later stages in the nodule, mainly in the two model legumes *M. truncatula* [72,110,111,112,113,114] and *L. japonicus* [115], but also in agronomically important species such as common bean [116,117], soybean [71,118,119] or chickpea (*Cicer arietinum*) [120,121]. 

Transcriptomic experiments are usually conducted using complex tissues or organs that participate in the symbiotic interaction (i.e., roots and nodules), which are formed by different cell types. This leads to the loss of valuable information about molecular responses that are spatially restricted or limited to specific types of cells. The potential to select the tissue or cell types used for transcriptomic studies in a more specific manner provides better and more accurate information of the cell-specific responses. Different techniques have been applied to separate epidermal from cortical responses within roots at early time points upon rhizobia inoculation, but also to distinguish the differential contribution of different zones of the nodule to gene expression. One simple method consists of the mechanical isolation of root hairs, the site where infection threads are initially formed. Characterization of the transcriptomic changes of the *M. truncatula* root hairs in response to *S. meliloti* and a mutant strain unable to produce Nod factor led to a comprehensive description of the reprogramming of these cells during early stages of the infection process [122]. This study showed that transcriptional changes in the epidermis were mainly associated with hormonal responses and the reactivation of the cell cycle. A similar approach in soybean allowed the identification of 1973 genes differentially expressed in root hairs in response to rhizobial infection [118]. More recently, Jardinaud et al. [123] combined laser capture microdissection (LCM) with RNA sequencing to characterize the reprogramming of gene expression induced by Nod factor in the root epidermis, highlighting the importance of cytokinin signaling and response in the nodulation process. 

Roux et al. also applied LCM followed by RNAseq to characterize gene expression across the zones that form the indeterminate nodules in *M. truncatula*: the apical meristem and the regions that contain the different stages of rhizobial colonization, from infection to fixation zones [124]. A similar approach was applied by Limpens et al. to isolate nodule cells at different developmental stages [125], providing a comprehensive description of changes in gene expression associated with non-infected cells in different nodule regions (nodule meristematic cells and non-infected cells from fixation zone), as well as to cells infected with rhizobia at different stages (from early infection to mature nitrogen-fixing cells). Both studies agreed that genes encoding proteins involved in cell divisions (e.g., CYCLIN A;1 and CYCLIN B3;1) and in the regulation of the root apical meristem (e.g., PLETHORA; SHORTROOT; SCARECRAW, WOX5, etc.) were enriched in the nodule meristematic zone. A set of genes required for DNA duplication (such as *CCS52A, CDC2* and *ORC2*, which control plant cell endoduplication), genes required for rhizobial infection (e.g., *ERN1*, *ERN2*, *EDF*) and genes involved in bacteroid differentiation (*DNF1*, genes encoding cystein-rich peptides and those involved in their maturation) were enriched in the proximal and distal infection zones of the nodule. Leghemoglobin and nodulin genes were enriched in infected cells of the fixation zone, whereas several genes involved in the synthesis of asparagine and in sucrose cleavage accumulated in non-infected cells of the fixation zone. 

Another technique that allows examining cell-specific expression is Translating Ribosome Affinity Purification (TRAP), in which the ribosomal protein L18 fused to the FLAG epitope is expressed under the control of tissue-specific promoters [126]. Immunopurification of polysomes (i.e., transcripts with two or more ribosomes) followed by RNAseq allows the characterization of the mRNA population associated with the translation machinery, which is known as the translatome [127]. Results obtained in our laboratory showed that not only RNAs coding for proteins involved in the Nod signaling pathway are dynamically recruited to polysomes but also non-coding RNAs, change their association with polysomes in response to rhizobial infection [128]. These studies characterizing changes at a global scale during nodulation in different cell types constitute a significant advance in the analysis of the transcriptome and promise to provide a more comprehensive understanding of plant cell reprogramming during root nodule symbiosis. 

Besides solid progress in our understanding of the signaling pathway activated by Nod factor perception, much less is known about the signaling triggered by other molecules involved in *Rhizobium* recognition. As was mentioned in previous sections, some of these markers of rhizobial identity can be crucial for the ecological outcome of the interaction, allowing plants to discriminate between different strains of a certain species with different symbiotic efficiencies. The partner selection theory proposes that plants have developed mechanisms to identify and select the best partners in a market scenario long before nodules are formed, and bacteria start to fix nitrogen [129]. This theory is consistent with the clonal selection that occurs during infections through the infection threads. In this way, symbiosis is stabilized, avoiding *cheating* by rhizobia that can enter the root cell to obtain benefits from the plant without reciprocating with efficient nitrogen-fixing reactions. Evidence supporting this model was obtained in common bean, a species that originated in America and was subjected to reproductive isolation to produce two different genetic pools, one in Mesoamerica and one in the Andean region. Genotypes from the Mesoamerican diversification center are able to select strains of *Rhizobium etli* that carry the allelic form α of the *nodC* gene in co-inoculation experiments, whereas this selectivity is not present in Andean accessions and cultivars [130,131]. In an attempt to identify the molecular mechanisms underlying the strain selectivity observed in Mesoamerican genotypes, a subtractive hybridization strategy was applied to identify candidate genes exhibiting differential expression when plants were inoculated with strains with different nodulation efficiency [132]. Forty-one candidate genes were accumulated at higher levels when a *R. etli nodC* α strain, which formed more nodules in co-inoculation experiments, was used in single inoculation experiments as compared with a strain that is outcompeted and prevalent in soils of the Andean region. A subset of these genes was further characterized by reverse genetics, revealing that transcription factors of the NF-Y family are implicated in the strain-specific response and the competing ability in co-inoculation experiments [109]. 

Another theory explaining how mutualism is stable over time is based on host-sanctioning once the symbiosis has been established [129]. In this view, plants can withdraw resources from cheating partners that do not fix nitrogen or are inefficient in the process. Theoretical data are supported by experimental evidence [133,134,135,136,137], although persistence of ineffective rhizobia once nodules are formed has been observed in different legume-rhizobia interactions, such as *L. japonicus* inoculated with *R. etli* [138], *Rhizobium tropici* or *Rhizobium* sp. NGR234 [139] or *M. truncatula* inoculated with *S. meliloti* [140]. In order to understand the differences in the molecular responses between symbiotic interactions with different rhizobial strains, transcriptomic studies have been applied to different combinations of host and rhizobia. Heath and collaborators [141] explored the nodule transcriptome by microarray analysis using two genotypes of *M. truncatula* combined with two *S. meliloti* strains. This analysis showed a stronger effect of the plant genotype as compared with the rhizobial strain. At the same time, the host determined the specific expression of approximately 25% of the *Rhizobium* genes. Interestingly, a bacterial transcription factor involved in the synthesis of the cell wall EPS succinoglycan was differentially regulated in both strains, suggesting that this molecule can be important in the establishment and maintenance of the interaction with specific plant genotypes. Burghardt et al. [142] also examined transcriptional changes of nodules to study the molecular changes associated with the efficiency of the symbiotic interaction. They used four *M. truncatula* genotypes combined with *S. meliloti* or *Sinorhizobium medicae*, covering different nodulation phenotypes and plant growth associated with the bacterial strain. As in the previous study, they found evidence of a strong influence of the host genotype on transcriptional changes observed in the plant, with a less pronounced effect of the symbiont and the host x symbiont combination. These results are important to understand the causes and consequences of the symbiotic outcome in terms of the trade-off between both partners.

Several transcriptomic studies have described the molecular responses triggered by signal molecules present in the surface or secreted by rhizobia, such as the Nod factor, cyclic glucan, LPS and EPS [53,112,113,117,143]. The presence of the succinoglycan of *S. meliloti* is required for normal infection, which depends on the induction of genes involved in protein metabolism and nodulins and the suppression of defense responses [53]. Another study showed that genes involved in meristem differentiation and cytokinin synthesis are also controlled by the presence of the EPS and are required for an efficient infection process [113]. A transcriptomic study of common bean plants inoculated with mutant strains of *R. etli* defective in the synthesis of Nod factor, EPS or the LPS dissected the role of these molecules in the modulation of molecular responses that include transcription factors, signaling molecules and defense responses, but also genes involved in the control of circadian rhythms and posttranscriptional gene silencing mediated by small RNAs, highlighting physiological processes that have been less considered in the context of the efficiency of symbiotic interactions. Taken together, these studies comparing molecular responses between mutant rhizobial strains with the wild type counterparts have been extremely helpful to dissect the plant responses that determine an efficient interaction.

## 6. Concluding Remarks and Future Perspectives

Intense research over the past years has revealed that rhizobia utilize signal molecules of a distinct nature to suppress plant immunity triggered by PAMPs, which allow them to infect the root tissue and colonize the nodule. Perception and integration of these diverse signals by the plant at different stages of the interaction are crucial for the success and outcome of the symbiotic process. Legumes have a first layer of recognition that discriminates between pathogenic and benefic bacteria. At this early stage of the interaction, Nod factors and effector proteins play dual roles, suppressing defense responses and stimulating the nodulation signaling pathway. After Nod factor recognition, secondary signals such as LPS and EPS contribute to select particular strains of rhizobia by suppressing defense responses during initiation and elongation of infection threads. Also, plant-derived NCR peptides are major factors of post-infection specificity. In indeterminate nodules, once rhizobia have been internalized into symbiosomes, NCR peptides promote terminal bacteroid differentiation; however, depending on the legume genotype-rhizobium strain combination, some specific NCR peptides can also maintain bacterial viability. Understanding these multiple and sequential layers of host-specificity are crucial not only for the maintenance of symbiosis, but also to favor the selection of rhizobial strains that better reciprocate in terms of nitrogen fixation. 

During evolution, legumes have acquired mechanisms to avoid low-quality partners; however, this trait could have been lost during domestication of wild genotypes [144]. One of the most challenging aspects of the study of nitrogen-fixing symbiosis is to unveil the genetic mechanisms that different legume species have developed through evolution to select the best symbiotic partners in a complex market scenario, which would allow designing neo-domestication strategies that positively select those characteristics. Considering the enormous diversity of the legume family [145,146], it is required to extend symbiosis research to species that are not well represented by the model legumes selected by the scientific community. Indeed, *M. truncatula* is one of the few species that presents terminally differentiated bacteroids inside their nodules. On the other hand, *L. japonicus*, *P. vulgaris* and *G. max* form determinate nodules, which are relatively rare in legumes. Other species differ in the infection process, morphology and structure of the nodules, reflecting the particular evolution of the different tribes [146]. Understanding the strategies used by different legume species to discriminate and select the best partners in their natural habitat is important not only for the improvement of nitrogen fixation efficiency in legumes, but also to transfer the nitrogen-fixing capacity to non-legume crops. Biotechnological efforts in this direction will certainly contribute to more sustainable agriculture.
